# Isatis root polysaccharide promotes maturation and secretory function of monocyte-derived dendritic cells

**DOI:** 10.1186/s12906-020-03103-2

**Published:** 2020-10-07

**Authors:** Xuebing Wang, Zewen Chen, Tong Chen, Xiao Li, Shucheng Huang, Hao Wang, Chao Tong, Fang Liu

**Affiliations:** 1 College of Veterinary Medicine, Henan Agricultural University, Zhengzhou, 450000 Henan province People’s Republic of China; 2 Key Laboratory for Animal-Derived Food Safety of Henan province, Zhengzhou, 450000 Henan province People’s Republic of China; 3 Biotechnology Developing Center of Henan Academy of Sciences, Henan Academy of sciences, Zhengzhou, 450002 Henan province People’s Republic of China; 4 Wuhu Overseas Student Pioneer Park, Wuhu, 241006 China

**Keywords:** Monocyte-derived DCs, Isatis root polysaccharide, Pseudorabies virus

## Abstract

**Background:**

Pseudorabies virus (PRV) is an animal virus that is globally responsible for the high economic losses in the swine industry. Isatis root is a traditional Chinese medicinal herb that possesses immune-enhancing and antiviral properties. However, the molecular mechanisms underlying the effects of the active component of the isatis root polysaccharide (IRPS) extract on immature dendritic cells remain elusive.

**Methods:**

In this study, we investigated the molecular changes in primary porcine peripheral blood monocyte-derived dendritic cells (MoDCs) during PRV infection, using enzyme-linked immunosorbent assay (ELISA) and quantitative reverse transcription-polymerase chain reaction. Additionally, we studied the effect of IRPS on PRV-infected DCs.

**Results:**

The results showed that IRPS stimulated the maturation of MoDCs, induced IL-12 secretion, and downregulated IL-6 expression.

**Conclusions:**

Collectively, these results suggest that IRPS is a promising candidate for promoting maturation of DCs and enhancing their secretory potential after PRV infection.

## Background

Dendritic cells (DCs) play a critical role in linking innate and adaptive immunity by presenting foreign or malignant antigenic peptides to T lymphocytes [1, 2]. As a major immune surveillance cell, the DC expresses multiple pattern recognition receptors including Toll-like receptors (TLRs), Fc receptors, and C-type lectin receptors that recognize numerous pathogen-associated molecular patterns [3]. During virus infection and/or antigen recognition, DCs progressively mature and produce increasing amounts of cytokines, express costimulatory molecules, and present antigens via the major histocompatibility complex (MHC) class I and II molecules. DCs stimulate naïve CD8^+^ and CD4^+^ T lymphocytes to differentiate into cytotoxic T cells and helper T (Th) cells, respectively, by antigen presentation and CD28 co-stimulation; additionally, they can present antigens to B lymphocytes and induce B cell proliferation and differentiation [4]. DCs also mediate immunological tolerance that leads to unresponsiveness to pathogenic antigens. Moreover, the IL-10 released from DCs regulates T cell anergy by stimulating regulatory T cell differentiation that suppresses T cell responses, including proliferation and IFN-γ production [5]. DC maturation is critical in most adaptive immune responses and is induced by a variety of stimuli, such as pro-inflammatory cytokines, pathogen-associated molecular patterns, damage-associated molecular patterns, immune complexes associated with Fc receptors, and bacterial products [6].

Herbal medicines have been used as therapeutics for various types of diseases and have shown significant antimicrobial, anti-inflammatory, immune-stimulatory, and immune-modulatory activities. The dry roots of *Radix isatidis* have been used in China and Asia for thousands of years [7]. Isatis root polysaccharide (IRPS) is the active component of *R. isatidis* and is responsible for the plant’s antiviral effect, which is achieved via the modulation of TLR-3 signaling and other immune-promoting effectors, such as IL-2 and IFN-γ [8]. *Astragalus* polysaccharides upregulate the expression of CD86 and MHC-II on DCs, induce DC maturation, and enhance their antigen-presenting capacity [9]. *Achyranthes bidentata* polysaccharide-treated mouse bone marrow-derived DCs express more CD86 and CD11a, produce higher levels of IL-12, and exhibit enhanced T cell priming [10]. *Ganoderma atrum* polysaccharides facilitate activation and maturation of murine myeloid-derived DCs [11]. However, whether IRPS induces DC maturation remains to be elucidated.

Pseudorabies virus (PRV) is a swine alphaherpesvirus that causes Aujeszky’s disease; in many other mammalian species (pig, sheep, dogs, cats, or other domestic animals), the virus causes fatal infections similar to that found in piglets. PRV is responsible for mild latent infections in adult pigs that can be lethal in infected neonatal hosts [12]. Although humans are not the hosts of PRV, clinical endophthalmitis caused by PRV infection has been reported in human [13]. IRPS possesses antiviral activity; however, the effect of IRPS on porcine DCs infected by PRV is unknown. In this study, we have demonstrated the direct and indirect effects of IRPS on porcine DCs. This study contributes to the understanding of viral infections and the role of immune cells in alleviating symptoms, thereby paving the way for preventing or reducing the occurrence of these diseases.

## Methods

### In vitro stimulation of primary porcine MoDCs

Pig peripheral blood (20 mL) from Large White/Landrace hybrid pigs with heparin (Sigma Chemical co., St. Louis, MO) 1:1 was diluted in phosphate-buffered saline (PBS). Lymphocyte separation solution (5 mL) was added to 15-mL centrifuge tubes followed by the careful addition of 5 mL of diluted fresh blood to the surface of the separation solution. This was centrifuged at 1800×g for 20 min at room temperature. The gray-white cloud-like lymphocyte layer was gently removed using a 1-mL syringe and added into a fresh centrifuge tube containing 10 mL of PBS and centrifuged at 1400×g for 10 min. Once the red blood cells sedimented, the supernatant was discarded, and 5 mL of red blood cell lysate was added, incubated for 5 min at 37 °C, and centrifuged at 1200×g for 7 min. The supernatant was discarded, and the precipitate was washed twice with PBS. The cells were resuspended in complete RPMI-1640 medium at a concentration of 3 × 10^6^ cells/mL. Trypan blue staining was used to determine cell viability. The cells were cultured in 6-well plates for 3 h at 37 °C in a humidified incubator containing 5% CO_2_. Only the adherent monocyte-DC precursor cells were preserved. Complete medium (3 mL) containing 20 ng/mL granulocyte-macrophage colony-stimulating factor (GM-CSF) and 20 ng/mL IL-4 was added to each well. Culture medium was replaced every 12 h for 6 days.

### IRPS extraction

Isatis root was purchased from Henan Herbal Medicine Center Chain Co., Ltd. (Henan Herbal Medicine Center Chain Co., Ltd., zhengzhou, China). The root (400 g) was immersed in water for 4 h, after which it was distilled with 800 mL of absolute ethanol for 2 h. The ethanol was distilled off by heating with caution in a distillation heating device for 2 h at approximately 70 °C. Five volumes of water were added to the remaining liquid. The extracted liquid was filtered and centrifuged at 3000×g for 15 min. The liquid was concentrated to 1 g/mL using a vacuum rotary evaporator; ethanol was slowly added to the solution while stirring to a final concentration of 60%. After overnight incubation, the precipitate was further precipitated by adding absolute ethanol and repeating twice. The final alcohol concentration was adjusted to 80%, and the precipitate was then incubated overnight. The polysaccharide isolate was obtained after freeze-drying. A standard curve was prepared using the absorbance of glucose-free solution measured at 625 nm with a UV spectrophotometer. The polysaccharide content was calculated based on the average absorbance of the sample.

### PRV infection

The PRV MinA strain was conserved in the Henan Province Key Laboratory of Animal Food Safety. Approximately 5 × 10^5^ cells/mL MoDCs were added to 96-well plates and cultured at 37 °C in a humidified incubator containing 5% CO_2_. After the cells formed a dense monolayer, the medium was discarded, and the plates were washed once with PBS. The virus solution (0.1 mL) was added to each well in different dilutions (10^− 1^–10^− 9^). After incubating for 1.5 h at 37 °C, the virus solution was discarded, and the cells were washed twice with PBS. The infected cells were subsequently cultured in 0.1 mL RPMI-1640 medium. The 10^− 2^ to 10^− 9^ dilution of virus was used to calculate TCID_50_. We used the Reed-Muench methods to calculate TCID_50_. Cell Counting Kit-8 (Dojindo, Kumamoto, Japan) solution was added to each well according to the manufacturer’s protocol. After incubating the plate for 2 h, the OD_450_ was detected using the Tecan spark spectrophotometer (Tecan Group Ltd., Zurich, Switzerland). Inhibition percentage = (control group OD - virus group OD)/(control group OD - blank); Distance ratio = (percentage for > 50% inhibition - 50%)/(percentage for > 50% inhibition - percentage for < 50% inhibition); and Log_10_TCID_50_ = distance ratio × logarithmic difference in the dilution coefficient + logarithm of drug dilution corresponding to the 50% inhibition rate.

### Determination of the safe concentration of IRPS for MoDCs

IRPS was mixed with RPMI-1640 medium containing GM-CSF, IL-4, and increasing concentrations of MoDCs; four replicate wells were set for each concentration. Lipopolysaccharide (LPS)-positive (with complete RPMI-1640 containing 100 ng/mL LPS, GM-CSF, and IL-4) and negative control wells (with complete RPMI-1640 containing GM-CSF and IL-4) were set up at the same time. Finally, the 96-well plate was placed in a 37 °C incubator with 5% CO_2_ for 48 h. Before collection, 20 μL of Cell Counting Kit-8 (Dojindo, Kumamoto, Japan) reagent was added to each well and incubated for 2 h. Thereafter, OD_450_ was measured to indicate cell proliferation.

### Enzyme-linked immunosorbent assay (ELISA)

Cell culture medium was collected from PRV-infected cells at different timepoints and centrifuged at 1200×g for 7 min. ELISA kits for IL-6 and IL-12 were purchased from Ray Biotech (Ray Biotech, Norcross, GA). The assays were performed in triplicate (including negative controls) as per the kit protocol. Absorbance was measured at 450 nm using the Tecan spark spectrophotometer (Tecan Group Ltd., Zurich, Switzerland).

### Quantitative reverse transcription-polymerase chain reaction (qRT-PCR)

Cells were collected after different timepoints of PRV infection. Total RNA was isolated and reverse transcribed using the PrimeScript^@^ RT Kit with gDNA Eraser (Takara, Dalian, China). The cDNA was used to determine relative mRNA content via real-time PCR using SYBR® Premix Ex Taq™ II (Takara, Dalian, China). The qRT-PCR data were analyzed using the 2^-ΔΔCt^ method. Porcine PRL-19 was used as an internal reference. Table 1 shows the primers used.

**Table 1:** Primer sequence for genes

Name	GenBank accession no.	Forward primer(5′-3′)	Reverse primer(5′-3′)	Amplicon length
IL-1β	NM214055	GAAAGATAACACGCCCACC	GGAGTTTCCCAGGAAGACG	193
IL-6	M80258	CTTCAGTCCAGTCGCCTTCT	GCCAGTACCTCCTTGCTGTTT	234
IL-10	L20001	GACCAGATGGGCGACTTGTT	CACGGCCTTGCTCTTGTTTT	243
IL-12p35	NM213993	ATGACAACCCTGTGCCTTAG	TTCAGAGCCTGCATCAGC	158
IFN-γ	AY188090	TTCAAAGGAGCATGGATGTG	CTCTTCCGCTTTCTTAGGTTAG	196
TNF-α	JF831365	CCCCCAGAAGGAAGAGTTTC	GGCATTGGCATACCCACTCT	160
PRL-19	AF435591	AACTCCCGTCAGCAGATCC	AGTACCCTTCCGCTTACCG	147

### Statistical analysis

All statistical comparisons were performed using the SPSS 18.0 software (PASW Statistics, Chicago, USA). Student’s t-test was used to determine the difference between two groups. Data have been expressed as the mean ± standard deviation. *P*-values < 0.05 were considered statistically significant.

## Results

### IRPS promotes immature DC (imDC) maturation and function

**Fig. 1:**
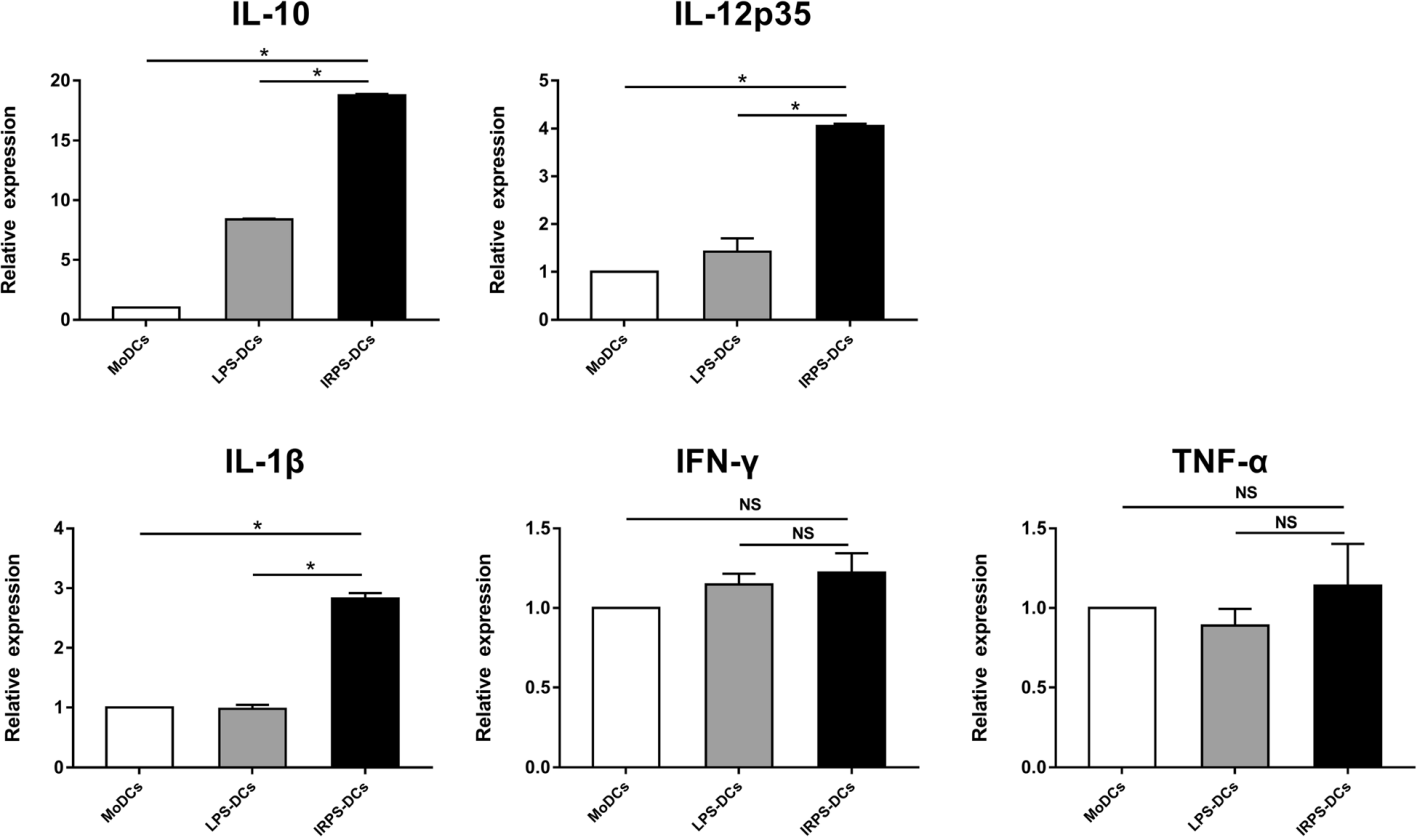
IRPS promote immature Dendritic cells mature and release cytokine factors. ImDCs means immature Dendritic cells, LPS-DCs means LPS-induced mature DCs and IRPS-DCs means IRPS-induced mature DCs. The experiment was independently repeated in triplicate

Before treating imDCs with IRPS, we determined that the safe concentration of IRPS for imDC cultures was 3.125 μg/mL (Table 2). IRPS showed a rate of inhibition of ~ 43.72%, indicating that it can promote imDC growth. To further investigate the effect of IRPS on imDCs, we detected the secretion of the cytokines IL-6 and IL-12 in IRPS-induced mature DCs using ELISA (Table 4 and Table 5). Interestingly, IL-12 secretion from IRPS-induced mature DCs was higher than that from LPS-induced mature cells or imDCs. The secretion of IL-6 from IRPS-induced mature DCs was lower than that from LPS-induced mature DCs or imDCs. The mRNA levels of IL-10, IL-1β, and IL-12p35 were higher in IRPS-induced mature DCs than in LPS-induced mature DCs or imDCs, whereas the mRNA levels of IFN-γ and TNF-α remained unchanged. Compared with imDCs, IL-10 mRNA levels increased by 18.77-fold in IRPS-induced mature DCs (Fig. 1). These findings suggest that IRPS induces DC maturation and enhances secretion by DCs.

**Table 2:** The toxicity of IRPS on imDCs

Group	Concentration (mg/mL)	Percentage of inhibition(%)
IRPS treatment	200	30.66
	100	29.02
	50	20.29
	25	−10.14
	12.50	−24.61
	6.25	− 38.32
	3.13	−43.72
	1.57	−19.65
LPS treatment	100 ng/ml	−24.64
Blank control		0

Note:The experiment was independently repeated four times for each concentration

### PRV directly infects imDCs

To obtain primary imDCs, we isolated porcine peripheral blood mononuclear cells and induced them in vitro by adding GM-CSF and IL-4. We used LPS treatment to boost imDC maturation. The mature/immature cells were infected with PRV (0.1 TCID_50_/cell). Copies of viral DNA in imDCs and LPS-induced mature DCs could be detected after 6 h of infection (Table 3). The number of virus particles in infected cells increased with longer infection and reached a peak at 48 h, after which there was no increase.

**Table 3:** The virus copy numbers after PRV infection

	ImDCs	LPS-DCs	IRPS-DCs
6 h	7.22 ± 0.05^a^	6.55 ± 0.16^aΔ^	7.30 ± 0.44^a^
12 h	7.98 ± 0.13^c^	7.86 ± 0.17^bc^	7.79 ± 0.08^b^
24 h	7.75 ± 0.04^b^	7.76 ± 0.07^b^	8.06 ± 0.11^bc^
36 h	8.13 ± 0.03^de^	8.12 ± 0.03^de^	8.23 ± 0.05^c^
48 h	8.16 ± 0.07^de^	8.23 ± 0.04^e^	8.39 ± 0.03^c^
60 h	8.21 ± 0.02^e^	8.20 ± 0.03^e^	8.33 ± 0.04^c^
72 h	8.04 ± 0.04^cd^	8.01 ± 0.04^cd^	8.22 ± 0.03^c^

Note:In the same group, shoulder marked with the different letter indicates significant difference. In the same time, Δ means *P* < 0.05 compared with imDCs and * means *P* < 0.05 compared with LPS-DCs. ImDCs means immature Dendritic cells, LPS-DCs means LPS-induced mature DCs and IRPS-DCs means IRPS-induced mature DCs. Number showed by lg (mean ± SD). The experiment was independently repeated four times

### PRV infection affects DC function

**Fig. 2:**
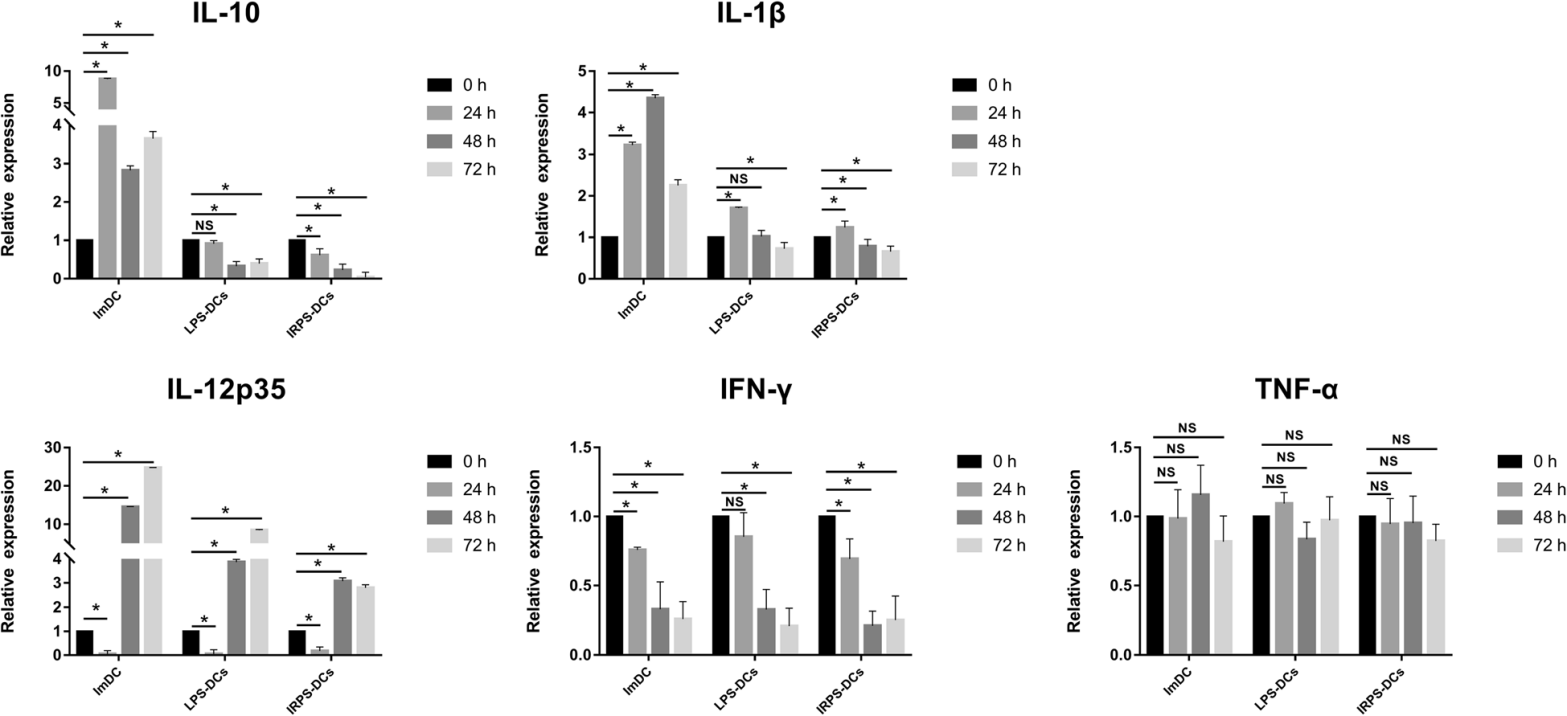
The relative expression of mRNA levels of IL-10, IL-12p35, IL-1β, IFN-γ and TNF-α in PRV infected DCs at different time (Comparison between different groups). The experiment was independently repeated in triplicate

The levels of IL-6 and IL-12, as observed via ELISA, in imDCs or mature DCs 24 h after PRV infection compared with 0 h, indicating a reduction in the secretion of IL-6 and IL-12 in the infected DCs (Table 4 and Table 5). After 72 h of PRV infection, the expression of IL-6 increased gradually; however, it was still lower than the level at 0 h. Moreover, we used qRT-PCR to quantify this expression in imDCs or mature DCs. The mRNA levels increased significantly after PRV infection. In the LPS-induced mature DCs, IL-12p35 mRNA levels increased 3.86- and 8.45-fold at 48 and 72 h, respectively. IL-1β mRNA level was upregulated by 1.72-fold at 24 h and downregulated after 48 h. IL-10 and IFN-γ were downregulated 24 h after PRV infection with no change in the expression of TNF-α (Fig. 2). Thus, this suggests that PRV inhibits secretion of TNF-α from mature DCs and reduces its immune function.

**Table 4:** The cytokine of IL-6 secreted by PRV infected cells

Infection time	ImDC	LPS-DCs	IRPS-DCs
0 h	60.40 ± 1.20^a^	63.79 ± 1.20^a^	43.46 ± 1.19^aΔ^*
24 h	40.93 ± 1.54^b^	15.64 ± 2.38^bΔ^	25.74 ± 2.38^bΔ^*
48 h	30.80 ± 2.39^c^	24.90 ± 5.96^bcΔ^	32.48 ± 2.39^bc^*
72 h	40.93 ± 2.39^b^	25.74 ± 4.77^cΔ^	37.55 ± 4.78^ca^*

Note:In the same group, shoulder marked with the different letter indicates significant difference. In the same time, Δ means *P* < 0.05 compared with imDCs and * means *P* < 0.05 compared with LPS-DCs. ImDCs means immature Dendritic cells, LPS-DCs means LPS-induced mature DCs and IRPS-DCs means IRPS-induced mature DCs. The experiment was independently repeated in triplicate

**Table 5:** The cytokine of IL-12 secreted by PRV infected cells

Infection time	ImDC	LPS-DCs	IRPS-DCs
0 h	278.11 ± 5.70^a^	312.05 ± 12.52^aΔ^	332.14 ± 16.00^aΔ^*
24 h	246.43 ± 3.42^b^	244.46 ± 3.42^b^	294.04 ± 3.49^bΔ^*
48 h	304.71 ± 3.05^c^	298.04 ± 3.46^c^	311.38 ± 12.01^b^
72 h	306.04 ± 1.99^c^	304.70 ± 10.26^c^	372.38 ± 7.59^c^

Note:In the same group, shoulder marked with the different letter indicates significant difference. In the same time, Δ means *P* < 0.05 compared with imDCs and * means *P* < 0.05 compared with LPS-DCs. ImDCs means immature Dendritic cells, LPS-DCs means LPS-induced mature DCs and IRPS-DCs means IRPS-induced mature DCs. The experiment was independently repeated in triplicate

### IRPS-induced mature DCs function better after PRV infection

**Fig. 3:**
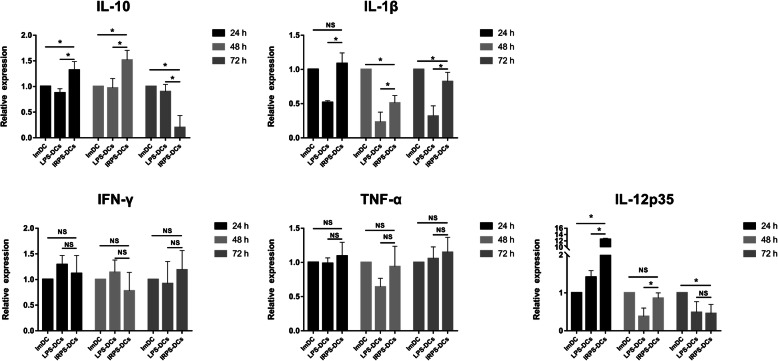
The mRNA levels of IL-10, IL-12p35, IL-1β, IFN-γ and TNF-α in PRV-infected MoDCs group, LPS-induced mature DCs group and IRPS-DCs means IRPS-induced mature DCs (Comparison between different infection time in one group). The experiment was independently repeated in triplicate

Since IRPS stimulated the maturation of imDCs, we investigated if IRPS functions better than LPS-induced mature DCs after PRV infection. The expression of IL-6 in LPS-DCs was higher than that in IRPS-DCs at 0 h. With increasing infection, the expression of IL-6 in LPS-DCs was lower than that in IRPS-DCs at 24, 48, and 72 h (Table 4 and Table 5). During PRV infection, the protein level of IL-12 in LPS-treated cells was lower than that in IRPS-treated cells. There was no difference in the mRNA levels of IFN-γ and TNF-α. However, qRT-PCR showed that the mRNA levels of IL-1β and IL-12p35 were higher in the IRPS-treated cells than in the LPS-treated cells. Interestingly, the IL-10 mRNA levels in the IRPS-treated cells were higher at 48 h but lower at 72 h (Fig. 3). These results indicate that IRPS enhances the secretory function of DCs better than LPS after PRV infection.

## Discussion

DCs are critical regulators of immune response, especially in the uptake and presentation of antigens and production of immune-related cytokines. Porcine mature DCs can be induced by GM-CSF, IL-4, and LPS. Additionally, we have found that they can be induced by IRPS. By quantifying the expression levels of IL-6, IL-12, and other factors, we demonstrated that IRPS induces imDC maturation. Through DC-mediated co-stimulation, T cells undergo proliferation and polarization [14]. Adaptive immune responses are primarily governed by cytotoxic T cells and Th cells that include several subsets with distinct functions [15]. Th1 and Th2 polarization is balanced by the stimulatory cytokines in the internal environment of co-stimulation [16]. When CD4+ T cells are primed by DCs, they reach an intermediate Th0 stage and polarize into Th1 in the presence of IL-12 and IFN-γ. Th1 cells have the ability to produce IFN-γ and TNF-α, thereby mediating cytotoxicity and pathogen elimination [17]. Th2 cells majorly mediate humoral immunity and anti-parasitic immune responses. In this study, we found that PRV infection induced the increase of IL-12 expression but decreased that of IL-6. Previous studies have shown that Kaposi’s sarcoma-associated herpesvirus infects DCs and impairs their function. Exposure of DCs to this infection results in an increased release of IL-6 and IL-10 with STAT3 phosphorylation. IL-6 and IL-10 promote immune suppression and inflammation [18]. IL-12 and IL-6 are Th1- and Th17-polarizing cytokines, respectively, in DCs. Jung et al. reported that IL-32γ stimulates the release of IL-12 from DCs and activates T lymphocytes toward the Th1 phenotype or secreted IL-6 and/or IL-1β activates T lymphocytes to the Th17 phenotype [19]. After stimulation by the herpes simplex virus, plasmacytoid DCs produce TNF-α, IFN-α, and IFN-γ, thereby triggering antiviral response and inducing CD4+ T cells to the Th1 phenotype [20]. IFN-γ produced by DCs can induce the type 1 or type 2 response. Mature DCs secrete a variety of inflammatory factors that are important in immune response [21, 22]. Although Th1, Th2, and Th17 cytokines may be produced at the same time, the cytokine profile bias depends on the pathogen and time of exposure. We did not study T-cell proliferation and T-cell activation after the DCs matured. Thus, the role of T cells after PRV infection needs to be understood in the future.

## Conclusions

IRPS is a promising agent for use in maturing DCs and enhancing IL-6 and IL-12 secretion. In comparison with LPS-induced mature cells after PRV infection, IRPS can improve the secretory potential of mature DCs.

## Data Availability

The datasets used and/or analyzed during the current study are available from the corresponding author on reasonable request.

## Abbreviations

PRV: Pseudorabies virus
IRPS: Isatis root polysaccharide
MoDCs: Monocyte-derived dendritic cells
DCs: Dendritic cells
MHC: Major histocompatibility complex
